# The Perceived Effectiveness of Cannabidiol on Adult Women with Inflammatory Bowel Disease

**DOI:** 10.3390/medicina60122059

**Published:** 2024-12-14

**Authors:** Ayelet Portman, Emily Bukovich, Janice Bissex, Molly Flanagan, Rachele Pojednic

**Affiliations:** 1 Department of Nutrition, College of Natural, Behavioral and Health Sciences, Simmons University, Boston, MA 02115, USA; 2 Department of Health and Human Performance, Norwich University, Northfield, VT 05663, USA; 3 Stanford Lifestyle Medicine, Stanford Prevention Research Center, Stanford University School of Medicine, Stanford, CA 94305, USA

**Keywords:** cannabidiol, inflammatory bowel disease, Crohn’s disease, ulcerative colitis

## Abstract

*Background and Objectives*: In this study we analyzed the perceived effectiveness of cannabidiol (CBD) in adult women with inflammatory bowel disease (IBD). *Materials and Methods*: We conducted an online cross-sectional survey which assessed disease severity and quality of life (QOL) in women with IBD (Crohn’s disease and ulcerative colitis) who used CBD versus those who did not. The survey included: the Harvey–Bradshaw Index, Partial Mayo Score, Short Inflammatory Bowel Disease Questionnaire, Bristol Stool Scale, and the Prime Screen Short Food Frequency Questionnaire. CBD utilization practices were also examined. STROBE reporting outcomes were followed. Statistical methods included Pearson and Spearman’s correlations and chi-square analyses. *Results*: Seventy-one (*n* = 71) women were included. Twenty-five (*n* = 25) were CBD users and forty-six (*n* = 46) were past/never users. Most current users ingested CBD once per day (40%), acquired CBD from a dispensary (56%), and utilized an oral tincture (40%) at a dose of 25 mg or less (56%). There was no statistical association between CBD use and disease severity, quality of life (QOL), fecal consistency, or food intake. However, CBD users reported decreases in IBD-related pain and nausea (76% and 64%, respectively), and improvement in appetite (60%). Overall, disease severity and QOL were inversely correlated (past/never users: *p* = 0.000, r = −0.544; current users: *p* = 0.042, r = −0.427). *Conclusions*: Women with IBD who use CBD insignificantly trended toward improved disease-related symptoms, appetite, and QOL compared to non-users. Disease severity and QOL were inversely associated, regardless of CBD use. These preliminary outcomes indicate the need for further research on CBD use in women with IBD.

## 1. Introduction

The use of cannabis and hemp-derived cannabinoids, like cannabidiol (CBD), have been increasing with the legalization of medical and recreational use throughout the United States [1]. There is growing interest in understanding the physiologic properties of constituents derived from cannabis and hemp as the use of crops continues to expand [2]. For example, CBD, one of the active components that can be extracted from both plants, is becoming increasingly popular as both a dietary supplement and an additive to functional foods [2]. While medicinal and recreational uses have become more popular, the evidence on mechanisms of action, effectiveness, and safety have lagged behind marketing to consumers. One particular concern regarding marketing strategies not backed by evidence are those utilizing cannabis- and hemp-derived products like CBD for relief of disease-related symptoms.

There are two main commercialized cannabinoids in the supplement market, CBD and tetrahydrocannabinol (THC), which are posited to achieve physiologic effects through the endocannabinoid system [3,4], although a mechanism of interaction between ligand and receptor is yet unclear. THC is the main psychoactive component in cannabis, while CBD has not been shown to have psychoactive effects but may exhibit health-related properties [3,4]. The endocannabinoid system is present throughout the human body, including the gastrointestinal (GI) tract, which has fueled interest in the impact of cannabinoids, particularly CBD, on GI-related diseases [3].

Currently, approximately 1.6 million Americans suffer from IBD—both Crohn’s disease (CD) and ulcerative colitis (UC)—and while prevalence does not differ between sexes, recent evidence shows the complex interaction between IBD and women’s physiology and psychology [5]. Women with IBD show worse psychological wellbeing and resilience than men, but they develop more escape and avoidance strategies to cope with the disease [5]. Cannabis and cannabinoid derivatives have grown increasingly popular for therapeutic use and symptom relief for IBD [6]. Preliminary data have indicated CBD may promote IBD symptom improvement [7], reduced steroid use [8], and increased appetite [9]. It is critically important to understand how women use and perceive these products.

While widely commercially available, there are very limited investigations into the effects of CBD as a component of treatment for GI-related diseases. Limited reports indicate that cannabis could be an alternative therapy for IBD, including CD and UC, and include a prospective cohort, retrospective observational studies, and three known randomized controlled trials [7,8,9,10,11,12,13]. There are only two known studies [11,12] primarily examining the perceived effectiveness of CBD alone in IBD patients. Furthermore, there are no known studies investigating women only. This is a gap in the literature, given the known sex differences in IBD symptomatology [5,14]. Since there is limited knowledge about the cause of IBD and no known cure, understanding complementary medical therapies is critical for increasing treatment options for those affected by the disease. It is particularly timely and important to understand how individuals with IBD use CBD, given the accessibility and marketing of commercially available products to this vulnerable population. The purpose of this study is to determine utilization practices and the perceived effectiveness of CBD in women with IBD.

## 2. Materials and Methods

This study utilized a cross-sectional online survey designed to understand CBD utilization practices and the perceived benefits of CBD use in women diagnosed with IBD. This study was considered exempt by the Simmons University Institutional Review Board. We also assessed study quality using the Strengthening the Reporting of Observational Studies in Epidemiology (STROBE) checklist.

### 2.1. Participants

Participants included a population of adult women diagnosed with IBD. Potential participants were provided study participation information via word of mouth and fliers in person in an urban area in the Northeastern United States through five dietitians who specialized in GI disorders and who may have had clients diagnosed with IBD. Participants were also recruited online through advertisement of this study on social media via snowball marketing on personal accounts as well as targeted postings in support groups for IBD and the Crohn’s and Colitis Foundation.

Participants were eligible to participate if they identified as female, were over the age of 18, not currently pregnant or nursing, had been diagnosed with IBD for at least six months, and had not been hospitalized because of their disease within the past month. Participants were included whether or not they used CBD, but were excluded if they used cannabis or THC. Participants completed an online screening form to determine eligibility, and, if deemed eligible to participate, they were asked to give consent to continue onto the online survey.

### 2.2. Survey

An anonymous online survey was administered between March and May 2020 via Qualtrics which included the following: demographic questions, novel questions to understand CBD utilization, and several validated tools—the Harvey–Bradshaw Index [15], the Partial Mayo Score [16], the Short Inflammatory Bowel Disease Questionnaire (SIBDQ) [17], and the Bristol Stool Scale [18]—to understand the perceived benefits of CBD utilization on disease symptoms and quality of life in the past one to two weeks. Dietary patterns over the prior 30 days were also considered by administering the Prime Screen Food Frequency Questionnaire (SFFQ) [19].

Demographic questions included the following: age, education level, alcohol and tobacco utilization, and menopausal status. Regarding CBD utilization, participants were asked if they believed there was a social stigma against people utilizing CBD/cannabis for medical purposes (yes/no) and if they believe legalization had improved the stigma (yes/no). Participants were then asked about their current CBD utilization (current, past, never). If participants were past/never users they were asked why they discontinued use or never began using.

Regarding dosage and administration, current and past CBD users were asked about their utilization practices. The frequency of utilization of CBD included five responses (one, two, or three times per day; once per week; twice per week; or open-ended “other”). The mode of administration questions included five options (smoking/inhalation, oral tincture, oral capsules, edibles, topical, or open-ended “other”). A question about the procurement of CBD included six responses (healthcare professional, dispensary, online order, retail store, grocery store or open-ended “other”).

The perceived effects of CBD were determined by comparing the outcomes of current users to past/never users based on answers to validated tools which assessed IBD status, pain relief, stool quality and frequency, disease activity, quality of life, and dietary patterns. Disease activity was determined using the Harvey–Bradshaw Index [15] (remission < 5, mild activity 5–7, moderate activity 8–16, severe activity > 16) and the Partial Mayo Score [16] (remission < 2, mild activity 2–4, moderate activity 5–7, severe activity > 7). While the Harvey–Bradshaw Index is indicated for CD and Partial Mayo for UC, these tools were answered by all participants regardless of disease state. Quality of life was indicated by SIBDQ [17] (score ranges from 10 to 70 with 10 indicating poor quality of life and 70 indicating high quality of life). Stool quality and frequency were determined using the Bristol Stool Scale [18] (types 1 and 2 indicate constipation, types 3 and 4 are healthy stool, while types 5–7 suggest diarrhea and urgency). Dietary patterns were determined using the Prime Screen Food Frequency Questionnaire (SFFQ) [19] (score ranges from 15 to 75 with a low score indicating a healthier diet and a higher score indicating a less healthy diet).

### 2.3. Statistical Analysis

Statistical analysis was performed using SPSS 25 (IBM Corp, Armonk, NY, USA). Descriptive statistics were used for demographic and utilization outcomes. Pearson correlations and Spearman’s correlations were applied to understand associations between continuous variables, and separated by CBD use. A chi-square and Student’s *t*-tests (two-tailed) were used to examine the relationships between variables and their group comparisons based on CBD use (i.e., current users versus past/never users). Outcomes were considered significant if *p* < 0.05. All continuous variables were assessed and confirmed for normality before they were analyzed.

## 3. Results

### 3.1. Demographics

Of the *n* = 119 participants who completed the online survey, *n* = 48 responses were not included in the analysis due to incomplete surveys. This resulted in a total of *n* = 71 participants, *n* = 25 of which were CBD users and *n* = 46 of which were past or never CBD users. Due to the time dependent nature of the surveys, past and never users were combined for analysis. Diagnoses of Crohn’s disease and ulcerative colitis were evenly distributed between both CBD users and past/never users (Table 1). Age range was 18–67 years and the majority (*n* = 65) had at least some college education. Ninety-two percent of CBD users expressed their agreement that legalization has made a difference in the stigma surrounding cannabis, compared to 69.6% among the non-users. Eighty-four percent of CBD users use additional complementary therapies compared to 65.2% of non-users. Race and ethnicity were not assessed.

### 3.2. Reasons for Never Using CBD or Discontinuing CBD Use

Past and never users were asked to provide a reason as to why they stopped or never used CBD as a treatment for their IBD symptoms. The majority of never users (48.3%) said they never used CBD because they were skeptical it would actually help with their symptoms while thirty-four percent (*n* = 10) were unsure where to buy a quality product. Additionally, twenty-seven percent (*n* = 8) did not use CBD because they were unsure whether it was legal, twenty-four percent (*n* = 7) were worried they would be drug tested and seventeen percent (*n* = 5) felt there was a stigma against CBD utilization that stopped them from using any products. The majority of past users (26.1%) said they discontinued their CBD use because of the cost (Table 2).

### 3.3. CBD Frequency/CBD Source

Most current CBD users ingested CBD once per day (40%) and the majority of participants reported that they acquired their CBD from a dispensary (56%). The majority of current users took CBD via an oral tincture (40%) at a dose of 25 mg of CBD or less (56%). The majority of past CBD users used an oral tincture (64.7%) at a dose of 10 mg of CBD (23.5%) or were unsure of the dosage used (23.5%) (Table 2).

### 3.4. IBD Symptoms

Seventy-six percent (*n* = 19) of current CBD users reported that CBD decreased IBD-related pain with twenty-four percent (*n* = 6) reporting no change. Sixty-four percent (*n* = 16) of current users experienced reduced IBD-related nausea with thirty-six percent (*n* = 9) reporting no change. Sixty percent (*n* = 15) of current users reported an improvement in appetite with forty percent (*n* = 10) reporting no change. No current CBD users reported negative effects for IBD-related pain, nausea, or appetite. Outcomes were further compared between current CBD users and past/never users (Table 3). There was no significant difference in any IBD symptom between groups; however, the comparison of mean outcomes indicated a lower disease severity and better quality of life among current users compared to past/never users.

**Figure 1:**
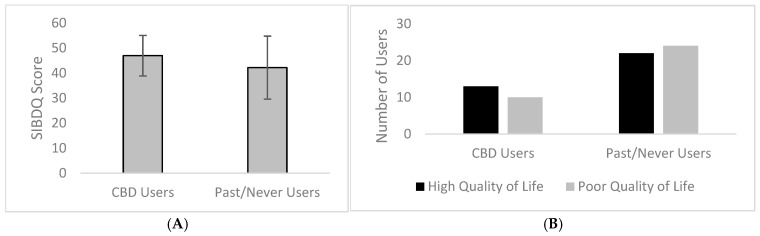
Short Inflammatory Bowel Disease Questionnaire (SIBDQ) scored higher in female users of cannabidiol (CBD) with inflammatory bowel disease (IBD) compared to non-users, indicating a decreased perception of severe symptoms across four domains: physical, social, emotional, and systemic and overall increase in quality of life. SIBDQ scores range from 10 to 70 with 10 indicating poor quality of life and 70 indicating high quality of life (**A**). More CBD users report high quality of life (≥45), while more past/never users report a poor quality of life (<45) (**B**).

Although not statistically significant, the Harvey–Bradshaw Index and Partial Mayo Score, which indicate a lower disease severity with a lower score, demonstrated lower scores overall among current versus past/never users (5.44 ± 4.1; 3.22 ± 2.1, respectively), although for all participants results were skewed toward lower scores overall. The SIBDQ, which indicates a higher quality of life with a higher score (range: 10–70), demonstrated that current users reported a higher score (46.96 ± 8.1) than past/never users (42.17 ± 12.6), although these differences were not significant (Figure 1). The Bristol Stool Scale, using bowel movement consistency to indicate disease severity, revealed that current users reported a slightly lower mean (4.36 ± 1.6) than never/past users (4.47 ± 1.7), indicating that both groups had moderately loose stools. The Prime Screen SFFQ assessed diet in the past year, taking into account vitamin use and the consumption of foods from various categories of nutrients. There was no significant difference in diet between groups, with each reporting scores that trended toward less healthy.

### 3.5. Relationships Between Symptoms and Diet

Among CBD users, there was a non-significant association between Prime Screen SFFQ and SIBDQ (*p* = 0.548; r = −0.139) and SFFQ and Partial Mayo Score (*p* = 0.838; r = −0.047). Among past/never users, there was a non-significant association between SFFQ and SIBDQ score (*p* = 0.429; r = −0.12), and a non-significant association between SFFQ and Partial Mayo Score (*p* = 0.875; r = 0.024).

With regard to dietary intake and disease severity, there was a non-significant association between SFFQ and Harvey–Bradshaw score (*p* = 0.384; r = −0.200) among CBD users, and a non-significant association between SFFQ and Harvey–Bradshaw score (*p* = 0.107; r = 0.241) among non-CBD users. There were statistically significant associations between disease severity (Harvey–Bradshaw and Partial Mayo) and quality of life (SIBDQ) indicating that a lower severity of disease is associated with a higher quality of life (Harvey–Bradshaw and SIBDQ current users: *p* = 0.000, r = −0.689; Harvey–Bradshaw and SIBDQ past/never users: *p* = 0.000, r = −0.840; SIBDQ and Partial Mayo current users: *p* = 0.042, r = −0.427; SIBDQ and Partial Mayo past/never users: *p* = 0.000, r = −0.544).

## 4. Discussion

The results of the present study indicated positive subjective trends between IBD symptoms and CBD utilization, although no associations were statistically significant. Of particular note, current CBD users reported a higher average quality of life, lower disease severity, decreased IBD-related pain and nausea, and improved appetite compared to non-users. Overall, participants believe that there has been a social stigma attached to the utilization of CBD/cannabis for medical purposes, but that the stigma is improving as legalization and recreational use grows.

Survey results demonstrated that the majority of current CBD users were taking a relatively low dosage of CBD (approximately 25 mg) by oral tincture; however, the sample size was small and the exact products used were unknown. While outcomes indicated CBD users perceived symptom improvement, the low dose could explain why CBD use did not demonstrate statistically significant differences in disease severity between users and past/never users. This is consistent with other investigations into commercially available doses of CBD. In a randomized clinical trial, in which participants received a 5 mg dose of CBD oil or a placebo, researchers found no significant difference between the study and placebo group [8]. It is possible that the commercially available dosages IBD patients are taking are too low to see a significant improvement in IBD-related symptoms [8].

It is important to note that current CBD users did report lower overall disease severity per the validated tools in this population, namely the Harvey–Bradshaw Index and Partial Mayo Score, when compared to the past/never CBD users. Naftali et al. also demonstrated a decrease in severity for the CBD group, and Irving and colleagues showed that Partial Mayo scores were lower for the group taking CBD [11,12]. Together, this growing body of evidence suggests that CBD use improves subjective symptom relief, although the specific mechanisms of action are yet to be elucidated.

The Bristol Stool Scale demonstrated no significant differences between CBD users and past/non-CBD users in stool consistency. Both groups indicated that stools were generally healthy, although trending toward diarrhea/urgency. This is a novel outcome, as the Bristol Stool Scale tool has not been utilized previously to understand the impact of CBD use in a population of individuals with IBD. These null findings could be a result of the low doses of CBD; however, future studies would be needed to support this outcome. Additionally, it is important to note that while CBD may not improve stool quality, it likely does not adversely affect it in this vulnerable population.

Lastly, with regard to symptoms, CBD use has been associated with a reduction in anxiety and improved quality of life in the general population [20]. This outcome has also been observed in individuals with IBD [11,12]. In the present study, CBD users reported a higher quality of life per the SIBDQ when compared to the past/never users, although statistically insignificant. It is possible that a reduction in anxiety due to CBD use, rather than direct symptom reduction in a population of individuals with chronic GI pain, could lead to improvements in the perception of quality of life. However, directionality should be considered, as it is also possible that those with lesser symptom severity tend to utilize complementary therapies with the belief that there will be a therapeutic effect. This should be an important area of distinction for future investigations of this cohort.

Finally, other studies have investigated the impact of whole cannabis (which includes CBD and THC, among other constituents) on overall IBD symptoms, and have demonstrated statistically significant improvements in IBD symptoms [9]. In an investigation conducted by Lal and colleagues, improvements in appetite and decreases in pain were found to be higher in those who used cannabis than those who did not use cannabis [9]. The present study, examining CBD use only, did find that the majority of participants who use CBD alone experienced a decrease in IBD-related pain and an improvement in appetite. However, that outcome failed to meet statistical significance. As such, it is conceivable that CBD alone is not sufficient for large symptom improvement in women with IBD.

### Strengths and Limitations

This study’s main strength is that it is the only known study to investigate the perceived effectiveness of CBD in adult women with IBD. The outcomes add critical value to the literature given that they address two current major gaps. First, in an ever-growing commercial market of CBD products [2], this study provides insight into a newly legalized substance widely marketed to and used by women. While it cannot distinguish direct effects of particular dosages or product applications (i.e., oral vs. topical), it gives an indication into utilization practices in the commercial market by a vulnerable population. Second, it is becoming increasingly clear that there is a sex bias in nutrition and supplementation research [21] and this study directly adds to the data lacking in female subjects.

This study also has four significant limitations. First, interpretation and generalizability are limited by a small sample size, and the distribution between current users and past/never users is mildly disproportionate. Second, there was also a risk of bias in survey responses in that a small, self-selecting user cohort (i.e., women who have been impressed with the belief that CBD may have therapeutic utility) may simply be prone to the suggestion that CBD will deliver some sort of benefit. Third, the Harvey–Bradshaw Index showed that all participants’ disease severity was skewed towards remission, indicating that perhaps CBD was not successful in significantly relieving symptoms compared to non-CBD use because of the lower severity of the disease in the entire cohort. Lastly, due to the small sample size and variety of administration (i.e., oral vs. topical), this study did not achieve the power to perform comparisons between differing dosages and the frequency of administration between current CBD users, or to control for confounding variables (i.e., other pharmaceutical use). Particularly with combined pharmaceutical and complementary therapies, this should be an important area of investigation in the future.

## 5. Conclusions

This study demonstrated that if women with IBD use CBD, they tend to be well educated, take low doses via oral tincture, and indicated a potential beneficial effect. Specifically, trends suggested that CBD could lead to improvements in IBD-related pain and nausea, increased appetite, and overall increased quality of life in this understudied and vulnerable cohort. Although self-reported outcomes were promising, no associations with CBD use were shown to be statistically significant. Additional investigations that include larger sample sizes, more variation among disease severity, and an interventional study design could further elucidate the results currently indicated.

## Figures and Tables

**Table 1:** Demographic and clinical characteristics of a group of (*n* = 71) women with inflammatory bowel disease.

	Current CBD Users	Past/Never CBD Users
	(*n* = 25)	(*n* = 46)
	*Mean ± SD*	
Age, y	31.83 ± 12.1	34.36 ± 13.1
	*Frequency n (%)*	
IBD Diagnosis		
Crohn’s Disease	12 (48%)	23 (50%)
Ulcerative Colitis	13 (52)	23 (50%)
Postmenopausal	2 (8%)	8 (17.4%)
Tobacco Use		
0 times	20 (80%)	41 (89.1%)
1–2 times per week	2 (8%)	0 (0%)
3–4 times per week	0 (0%)	1 (2.2%)
Daily	3 (12%)	4 (8.7%)
Alcohol Use		
0 times	10 (40%)	27 (58.7%)
1–2 times per week	11 (44%)	16 (34.8%)
3–4 times per week	3 (12%)	2 (4.3%)
Daily	1 (4%)	1 (2.2%)
Education Level		
Less than high school	0 (0%)	0 (0%)
High School	1 (4%)	5 (10.9%)
Some College	4 (16%)	10 (21.7%)
College	12 (48%)	26 (56.5%)
Master’s Degree	7 (28%)	4 (8.7%)
Doctoral or Professional Degree	1 (4%)	1 (2.2%)
Stigma *		
Yes	22 (88%)	37 (80.4%)
Legalization **		
Yes	23 (92%)	32 (69.6%)
Prescription Medication		
Yes	18 (72%)	36 (78.3%)
Complementary Therapy		
Yes	21 (84%)	30 (65.2%)

* Do participants believe that there is a social stigma against people using CBD/cannabis as a medical treatment? ** Has legalization made a difference in the stigma surrounding CBD/cannabis?

**Table 2:** Administration and dosage information for adult women with inflammatory bowel disease who are currently using CBD (*n* = 25) or previous used CBD (*n* = 17).

	Current CBD Users (*n* = 25)	Past CBD Users (*n* = 17)
	*n* (%)	
Current CBD Source		
Healthcare Professional	1 (4)	
Dispensary	14 (56)	
Online	6 (24)	
Retail Store	1 (4)	
Grocery Store	1 (4)	
Other	2 (8)	
Frequency of Current Use		
Once per day	10 (40)	
Two times per day	6 (24)	
More than three times per day	3 (12)	
Once per week	1 (4)	
Twice per week	1 (4)	
Other	4 (16)	
Mode of Administration		
Smoking/Inhalation	7 (28)	2 (12)
Oral Tincture	10 (40)	11 (65)
Oral Capsules	4 (16)	1 (6)
Edibles	1 (4)	2 (12)
Topical	1 (4)	1 (6)
Other	2 (8)	0 (0)
Dosage (mg)		
5	2 (8)	2 (12)
10	5 (20)	4 (23.5)
25	7 (28)	2 (12)
50	3 (12)	2 (12)
>50	2 (8)	0 (0)
Unsure	5 (20)	4 (23.5)
Other	1 (4)	3 (17.5)
Reason for Discontinued Use		
Adverse Effect		1 (2.2)
Did not Help		6 (13)
Price		12 (26.1)
Drug Testing		3 (6.5)
Stigma		2 (4.3)
Legality		0 (0)
Other		2 (4.3)

**Table 3:** Disease activity status of (*n* = 71) adult women with inflammatory bowel disease (IBD) either currently using CBD or who are past/never CBD users using five validated tools *.

	Current CBD Users (*n* = 25)	Past/Never CBD Users (*n* = 46)	*p*-Value
	Mean ± SD		
Harvey–Bradshaw Index ^1^	5.44 ± 4.1	6.96 ± 5.3	0.14
Partial Mayo Score ^2^	3.22 ± 2.1	3.61 ± 1.8	0.11
SIBDQ ^3^	46.96 ± 8.1	42.17 ± 12.6	0.43
Bristol Stool Scale ^4^	4.36 ± 1.6	4.47 ± 1.7	n/a
Prime Screen SFFQ ^5^	40.19 ± 5.9	40.15 ± 5.88	0.98

^1^. Harvey–Bradshaw Index [13]: Remission < 5, mild activity 5–7, moderate activity 8–16, severe activity > 16. ^2^. Partial Mayo Score [14]: Remission < 2, mild activity 2–4, moderate activity 5–7, severe activity > 7. ^3^. Short Quality of Life Questionnaire for Inflammatory Bowel Disease (SIBDQ) [15]: score ranges from 10 to 70 with 10 indicating poor quality of life and 70 indicating high quality of life. ^4^. Bristol Stool Scale [16]: score ranging from type 1 to 7 (solid to liquid stools). ^5^. PRIME Screen SFFQ [17]: score ranges from 15 to 75 with a low score indicating a healthier diet and a higher score indicating a less healthy diet. * Statistics were conducted with Student’s *t*-test (two-tailed) and outcomes were considered significant if *p* < 0.05.

## Data Availability

All data can be made available upon request to the corresponding author.
